# Cell-mediated immune responses of beta-glucan on the pathogenesis of chronic tuberculosis infection

**DOI:** 10.3389/fimmu.2026.1784128

**Published:** 2026-04-29

**Authors:** Shreya Chakraborty, Devi Rajeswari V

**Affiliations:** Department of Biomedical Sciences, School of Biosciences and Technology, Vellore Institute of Technology, Vellore, Tamil Nadu, India

**Keywords:** beta-glucan, biomarkers, immune system, pathogenesis, T-lymphocytes, tuberculosis

## Abstract

Tuberculosis (TB) has become the most common reason for contagious disease-related fatalities globally. Due to the estimated 8.6 million fresh tuberculosis infections and 1.6 million fatalities, this disease is a leading contributor to mortality globally. Tuberculosis refers to a condition in which individuals are infected with *M. tuberculosis* but do not exhibit clinical symptoms and are generally not capable of transmitting the disease. β-glucan, a biologically active polysaccharide known for its immunomodulatory properties, has recently gained attention for its role in regulating metabolic and immune responses. Through mechanisms such as trained immunity, macrophage activation, and cytokine modulation, β-glucan may enhance host defense against *M. tuberculosis* and influence susceptibility to tuberculosis disease progression. Hence, this study focuses on the underlying relationship of beta-glucan involved in Tuberculosis infection, along with their physiopathology, and, clinical implications including the immunological responses.

## 1 Introduction

Tuberculosis (TB) is characterized as a persistent inflammatory contagious disease with significant socioeconomic repercussions brought on by the bacillus *Mycobacterium tuberculosis.* Globally, TB is a leading reason of mortality with an estimated 8.6 million fresh infections and 1.6 million fatalities per year (1). After coronavirus tuberculosis (TB) became the next most prevalent infectious agent-related reason of mortality worldwide in 2022, accounting for nearly twice as many fatalities as HIV/AIDS. A total of approximately 1.30 million deaths were related to tuberculosis worldwide in 2022. 7.5 million individuals had a new TB diagnosis and were formally reported as cases worldwide in 2022 (2).

Transmission of *M. tuberculosis* takes place by inhaling particles of such bacteria that are excluded from the mucus of a person with a current illness (1). About 90–95 percent of the time, *M. tuberculosis* transmission is medically asymptomatic and not contagious; and such condition is known as tuberculosis (TB) infection, which indicates the presence of immunological evidence of infection without clinical signs or radiological manifestations of active disease. It is estimated that nearly one-quarter of the global population carries *M. tuberculosis* infection; however, only about 5–10% of infected individuals progress to active tuberculosis disease during their lifetime, depending on host immune status and other risk factors (3). Significant alterations in a patient’s immunological condition might increase TB initiation or recurrence. The symmetry involving the microbe and the host’s defense determines how quickly the disease infection progresses (4). Even though the immune system manages the disease, sterilization is not always the result of this management. When *M. tuberculosis* is identified in the vesicles of macrophages, the connection between these host cells and the T cells is crucial for the preventive immune activity towards the mycobacteria (5). In the past few years, the frequency of TB has decreased in high-income nations, but it has not decreased in nations with significant instances of HIV infection, malnutrition, cramped living circumstances, or inadequate facilities for tuberculosis management.

Though the link between tuberculosis with other diseases along with its co-morbidities is a bit unclear as little knowledge has been found for their molecular linkage, and the investigation is under research. For each of these disorders, several artificially synthesized medications have a variety of negative effects on the human body (6). As a result, organic, non-synthetic, organically existing nutritional supplements have become more important over the past few decades for maintaining wellness, preventing the onset of illness, and lowering the likelihood of adverse outcomes of these conditions as well as their associated consequences.

β-glucans are naturally occurring polysaccharides composed of glucose units linked primarily through β-(1→3) and β-(1→6) glycosidic bonds. They are structural components of the cell walls of fungi, yeast, bacteria, and certain cereals such as oats and barley. Due to their immunomodulatory properties, β-glucans have attracted significant attention as biological response modifiers capable of enhancing host defense against infectious diseases. In the context of tuberculosis, β-glucans are being explored as potential host-directed therapeutic agents that may support immune responses against *M. tuberculosis*. Because of their distinctive molecular composition and specific functions related to the control of an individual’s metabolic processes and because of their distinctive features, beta-glucans have gained a lot of popularity nowadays. These substances have an immune-modulating effect that can activate macrophages, phagocytose the infectious agent, and produce proinflammatory cytokines, all of which can stimulate immunological action (7). They change the gut microbiome especially, which has a good impact on the organism’s stability. The ability of beta-glucans to stimulate the immune system has a wide range of medicinal applications like the treatment of a variety of long-term inflammatory illnesses that can benefit from its capacity to modify humoral and cellular responses (8).

## 2 Global epidemiology of tuberculosis infection

Tuberculosis (TB) remains a major global health burden, with approximately 10.6 million cases reported in 2021, with an increase in 4.5% compared to 2020. Despite widespread exposure, only 5–10% of infected individuals progress from latent infection to active disease, highlighting the critical role of host immune responses in determining disease outcome (9). Variability in TB epidemiology is strongly influenced by host immune competence rather than exposure alone. Conditions such as HIV infection (10), diabetes mellitus (11, 12), malnutrition (13), and other immunocompromised states increase susceptibility to active tuberculosis by impairing innate and adaptive immune responses. Emerging evidence suggests that innate immune memory, or trained immunity, plays an important role in early control of *M. tuberculosis* (14–17). β-glucan, a well-established inducer of trained immunity, enhances macrophage responsiveness, cytokine production, and antimicrobial activity (18). Although direct epidemiological evidence linking β-glucan exposure to reduced TB incidence remains limited, its immunomodulatory effects provide a mechanistic basis through which trained immunity may influence disease susceptibility and progression.

### 2.1 Major risk factors for progression to active tuberculosis

**Figure 1:**
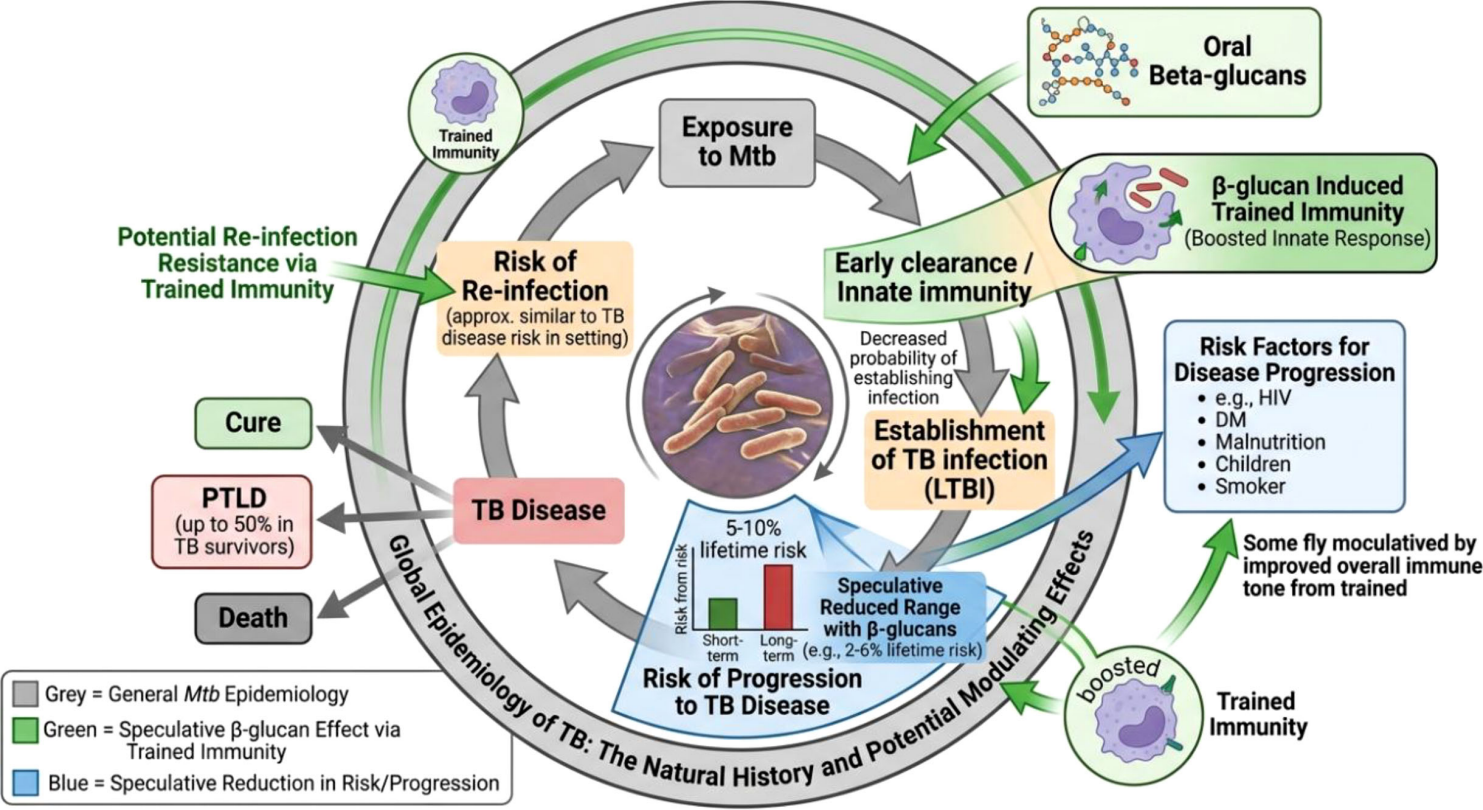
Global epidemiology of Mycobacterium tuberculosis (Mtb) and potential modulation by β-glucan–induced trained immunity. The diagram outlines the progression from exposure to latent infection and active disease, highlighting the 5–10% lifetime risk of disease development influenced by host factors such as HIV, diabetes, and malnutrition. β-glucans (green) are shown as potential modulators that enhance innate immune responses through trained immunity, improving early pathogen clearance and potentially reducing progression to active tuberculosis.

Several biological and host-related factors influence the likelihood of progression from tuberculosis infection to active disease (5). Close contact with individuals suffering from pulmonary bacilliferous tuberculosis remains the primary determinant for the transmission of *M. tuberculosis*. Following exposure, a large proportion of individuals develop tuberculosis infection without clinical symptoms. It is estimated that nearly one-quarter to one-third of the global population carries latent or asymptomatic tuberculosis infection, although only a minority progress to active disease. Approximately 5% of infected individuals develop active disease within the first year after infection, while an additional 5% may develop disease later during their lifetime (4). Host-related comorbidities are strongly associated with increased tuberculosis risk. Individuals with HIV infection, diabetes mellitus, chronic kidney disease, malnutrition, and other metabolic disorders exhibit impaired immune responses that facilitate the progression from infection to active tuberculosis disease. In particular, immune dysfunction resulting from chronic diseases significantly affects macrophage activation, cytokine signaling pathways, and cell-mediated immunity required for effective containment of *M. tuberculosis* (11, 12). These observations highlight that progression to active tuberculosis is primarily driven by alterations in host immune competence rather than exposure alone. Impairment of innate immune responses, particularly macrophage function and cytokine production, plays a central role in disease susceptibility. In this context, modulation of innate immunity through trained immunity has emerged as a potential mechanism influencing tuberculosis outcomes (14). β-glucan, a potent inducer of trained immunity, enhances macrophage responsiveness, cytokine production, and antimicrobial activity (18). Through epigenetic and metabolic reprogramming of innate immune cells, β-glucan may improve early containment of *M. tuberculosis* and reduce the likelihood of progression to active disease. Thus, integrating immunological risk factors with trained immunity provides a more relevant framework for understanding tuberculosis susceptibility and supports the potential role of β-glucan–based host-directed therapeutic strategies (**Figure 1**).

## 3 Pathophysiology of mycobacterium tuberculosis infection and progression to active tuberculosis disease

Following inhalation of *M. tuberculosis*, individuals may develop tuberculosis infection, characterized by immunological evidence of infection without clinical symptoms. In most cases, the immune system contains the bacteria within granulomas, resulting in an asymptomatic state. However, in some individuals with weakened immunity or risk factors, the infection may progress to active tuberculosis, marked by bacterial replication, tissue damage, and clinical symptoms (19, 20). Almost all cases of tuberculosis are transmitted from one person to another, and interaction with a donor illness case is necessary for infection. Although smear-negative/culture-positive patients can cause up to 17% of fresh infections, exposures to sputum smear-positive infections cause more than 80% of fresh TB instances (21). Even though little new research shows that both innate and adaptive immune mechanisms could be impacted, the immunological reason for elevated vulnerability in people with Tuberculosis is still largely undefined (22). Numerous authors concur that the incidence of pulmonary tract disorders is increased by decreased cell-mediated immunity and neutrophil granulocyte malfunction (23). A modified CD4/CD8 lymphocyte balance, abnormalities in cytotoxic activity, phagocytic activity, and granulocytic cell functioning, a reduction in extracellular monocytes with reduced phagocytosis, and elevated concentrations of type 1 and type 17 cytokines are all signs of immune insufficiency, which is common in TB (24).

This enhanced sensitivity to Tuberculosis may be caused by malfunctioning and systemic inflammation, which are frequently observed (25). Individuals with Tuberculosis who are malnourished and inactive simultaneously trigger the stress hormones cortisol, glucagon, and adrenaline. Research demonstrates that lung excision in individuals with pulmonary Tuberculosis lowered the incidence of Tuberculosis and improved following Tuberculosis therapy (26). Last but not least, vitamin A, C, and D deficits have also been linked to an increased risk of developing Tuberculosis (27).

It might be fascinating to concentrate on undefined genomic components to have a clearer knowledge of the connections between such disorders. The 11p13 locus, which is situated directly downward streaming of the Wilms’ tumour-1 gene, which controls the IL10 and vitamin D receptor mechanisms, has indeed been hypothesized to perform a part in the pathophysiology of both tuberculosis despite there being no close correlation with any particular genes or gene activities to date (28). Tuberculosis vulnerability appears to be mediated by two additional genes, HK2 and CD28. Numerous potential sites have indeed been connected explicitly to Tuberculosis, such as the tuberculosis risk site on chromosome 18q11.1, and many other loci that have been found strongly connected to immunological signaling genes. Nevertheless, no common loci have yet been discovered, except for the vitamin D receptor (VDR), which has been widely studied in relation to tuberculosis susceptibility due to its role in macrophage activation and antimicrobial peptide expression (29–32). Several genome-wide association studies have also identified susceptibility loci on chromosomes such as 18q11.2 and genes involved in immune signaling pathways, supporting the role of host genetic variation in tuberculosis pathogenesis (33–35). MiRNAs, a group of small RNAs that do not codify and control genomic activity in a variety of both normal and abnormal cellular processes, may also serve other purposes. Increasing research points to certain miRNA suppression by mycobacteria, which might serve as a representation of their pathogenicity traits and serve as disease markers, even if the role of miRNA in the relationship with Tuberculosis is not entirely known (36).

## 4 Immunomodulatory mechanisms of beta-glucan in tuberculosis

### 4.1 Trained immunity and epigenetic reprogramming

β-glucan has emerged as a potent inducer of trained immunity, a process through which innate immune cells undergo long-term functional reprogramming following an initial stimulus (37, 38). Unlike classical adaptive immunity, trained immunity enhances the responsiveness of monocytes and macrophages through epigenetic and metabolic modifications, thereby promoting improved protection against subsequent infections, including *M. tuberculosis* (Mtb) (39).

Experimental evidence demonstrates that β-glucan exposure induces histone modifications at promoter regions of pro-inflammatory genes in human monocytes, leading to enhanced transcriptional readiness (40–42). This epigenetic remodeling is associated with increased production of cytokines such as IL-1β, TNF-α, and IL-6 upon secondary stimulation with mycobacterial antigens, along with suppression of Mtb growth (40–42). IL-1 signaling has been identified as a critical mediator of β-glucan–induced protection against Mtb infection (43, 44), highlighting its central role in host-directed immune modulation. The enhanced cytokine production after β-glucan exposure reflects trained immunity rather than temporary inflammation. Studies show that β-glucan can reprogram bone marrow progenitor cells, leading to sustained generation of primed monocytes and macrophages with enhanced antimicrobial activity through lasting epigenetic and metabolic changes. Importantly, β-glucan not only acts on mature circulating monocytes but also reprograms hematopoietic stem and progenitor cells within the bone marrow (40). This reprogramming results in enhanced myelopoiesis and the generation of functionally superior macrophages and granulocytes (39, 40). Such long-term modulation provides a mechanistic explanation for the sustained anti-infective effects of β-glucan despite the relatively short lifespan of circulating myeloid cells. In the context of tuberculosis, this trained phenotype may contribute to improved early containment of Mtb and reduced bacterial proliferation (39). Although β-glucan–induced trained immunity is well demonstrated in *in vitro* and *in vivo* studies, its translation to human tuberculosis remains limited. Most studies rely on monocyte stimulation or simplified infection models that do not fully reflect the complex granulomatous environment of human TB. Moreover, variations in β-glucan sources, structures, and dosing hinder direct comparison between studies, emphasizing the need for standardized experimental approaches and clinical validation.

### 4.2 Receptor-mediated recognition and signaling pathways

**Figure 2:**
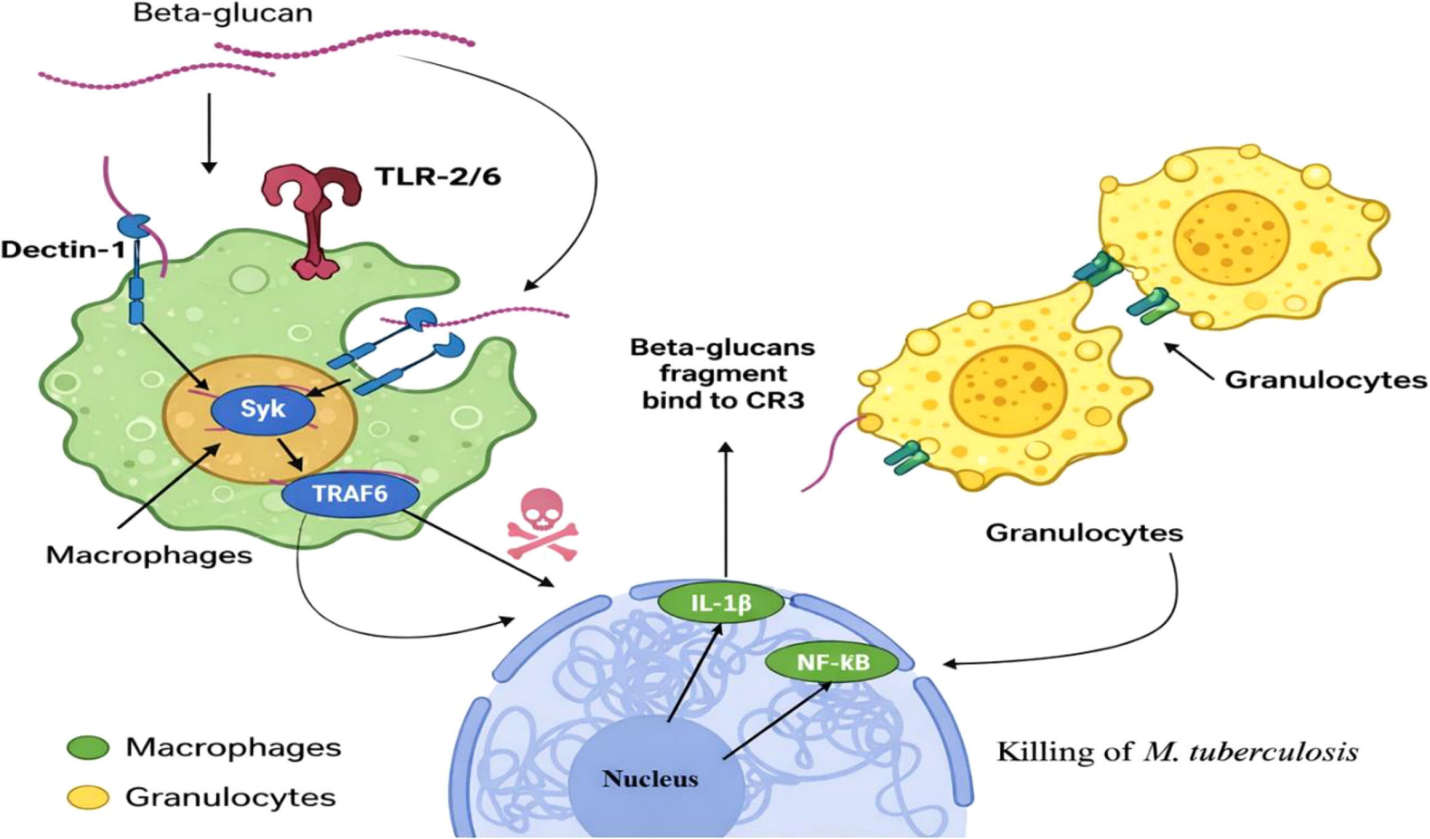
Mechanistic illustration of β-glucan–mediated immune activation in tuberculosis. β-glucan are recognized by pattern recognition receptors (PRRs) such as Dectin-1 and TLR-2/6, triggering an intracellular signaling cascade involving Syk and TRAF6. This leads to the nuclear translocation of NF-κB and the secretion of pro-inflammatory cytokines like IL-1β, which are critical for the intracellular killing of M. tuberculosis. Simultaneously, β-glucan fragments bind to the CR3 receptor on granulocytes, priming these effector cells to target tumor or infected cells through interaction with iC3b-opsonized monoclonal antibodies.

The immunological effects of β-glucan are initiated through its recognition as a pathogen-associated molecular pattern (PAMP) by pattern recognition receptors (PRRs) expressed on innate immune cells (45, 46). Among these receptors, Dectin-1 is considered the principal β-glucan receptor (47–50). Additional receptors—including complement receptor 3 (CR3), lactosylceramide, scavenger receptors, and Toll-like receptors (TLR-2, TLR-4, and TLR-6)—contribute to signal amplification and immune coordination (45–49). Upon binding to Dectin-1 on macrophages and dendritic cells, β-glucan activates intracellular signaling cascades involving Syk kinase and NF-κB pathways (**Figure 2**), resulting in transcription of pro-inflammatory mediators (47, 51). Dectin-1 contains an intracellular ITAM-like domain that becomes phosphorylated after β-glucan binding, recruiting Syk and activating the CARD9–Bcl10–MALT1 complex. This triggers NF-κB and MAPK signaling, leading to expression of inflammatory and antimicrobial genes. These pathways also promote metabolic reprogramming of monocytes via mTOR and HIF-1α, supporting glycolysis and cytokine production associated with trained immunity (52, 53). Importantly, these immunometabolic effects may be particularly relevant in individuals with diabetes, who are at increased risk of developing tuberculosis. Hyperglycemia can suppress macrophage activation and reduce cytokine production, compromising the host’s ability to control *M. tuberculosis*. β-Glucans have been shown to induce immunometabolic reprogramming through activation of the mTOR and HIF-1α pathways, promoting glycolysis and enhanced production of pro-inflammatory cytokines such as IL-1β. This metabolic shift supports trained immunity and may help restore antimicrobial functions in immune cells, thereby potentially reducing susceptibility to tuberculosis in individuals with diabetes. Crosstalk between Dectin-1 and TLR signaling further refines the immune response by balancing pro- and anti-inflammatory cytokine production (46, 54). This coordinated signaling enhances phagocytosis, antigen presentation, and microbial killing—processes essential for controlling Mtb infection (55, 56). CR3 also recognizes soluble β-glucan fragments and promotes opsonophagocytosis. Its activation enhances cytoskeletal rearrangement and facilitates pathogen engulfment by macrophages and neutrophils, supporting antimicrobial defense during infection (57). Following internalization, β-glucan particles are processed within macrophages and distributed through the reticuloendothelial system and bone marrow (42, 48). Smaller β-glucan fragments may be taken up by granulocytes and monocytes via CR3, further propagating immune activation (42, 48). Through these receptor-mediated pathways, β-glucan strengthens both innate and adaptive immune interfaces, supporting T-cell activation and improved host defense mechanisms against tuberculosis (45, 51). Despite extensive characterization of Dectin-1 signaling, the contribution of different PRRs in tuberculosis remains unclear. Evidence suggests possible synergy between Dectin-1 and TLR pathways, but conflicting cytokine responses indicate that receptor activation may depend on β-glucan structure and host immune status. Further mechanistic studies are needed to clarify the receptor-specific pathways underlying β-glucan–mediated protection against *M. tuberculosis*.

### 4.3 Functional immunological outcomes and therapeutic implications

The downstream immunological consequences of β-glucan signaling are particularly relevant to tuberculosis pathogenesis. Enhanced macrophage activation promotes improved phagocytosis and intracellular killing of Mtb (55, 56). This enhanced phagocytic activity involves cytoskeletal rearrangement and increased expression of phagocytic receptors, enabling macrophages to more effectively engulf mycobacteria. β-glucan also promotes phagosome maturation and phagolysosome fusion, restoring intracellular pathways often inhibited by *M. tuberculosis* and improving bacterial clearance (58). Increased production of IL-1β and TNF-α supports protective Th1-type responses, which are critical for granuloma formation and bacterial containment (43, 44). In addition to IL-1β and TNF-α, β-glucan also promotes the production of IL-6 and IL-12, which support Th1 responses and IFN-γ–mediated macrophage activation. This cytokine production involves NF-κB–driven transcription of pro-cytokines and inflammasome activation (e.g., NLRP3), leading to caspase-1–mediated maturation of IL-1β, thereby enabling controlled inflammatory responses during mycobacterial infection (59, 60). Emerging evidence suggests that β-glucan may influence granuloma organization and maturation (61, 62). By promoting recruitment and functional optimization of immune cells within granulomatous structures, β-glucan could enhance the host’s ability to confine Mtb and limit dissemination (61, 62). Granuloma formation involves coordinated interactions between macrophages, T lymphocytes, and other immune cells to contain *M. tuberculosis*. β-glucan–induced cytokines such as TNF-α and IL-1β promote recruitment and activation of immune cells at infection sites, enhancing immune responses within granulomas and improving bacterial containment. Given that ineffective granuloma maintenance contributes to disease progression, modulation of granulomatous immunity represents a promising host-directed therapeutic strategy. Additionally, β-glucan has been explored as an adjunct to conventional anti-tuberculosis therapy (63). Experimental findings indicate that β-glucan administration may reduce bacterial burden and enhance clearance of Mtb when used alongside standard treatment regimens (63). Its ability to induce trained immunity parallels immunomodulatory effects observed with Bacille Calmette-Guérin (BCG) vaccination (42), suggesting potential utility as an immunological adjuvant to enhance anti-tuberculosis vaccines (43, 44).

Collectively, β-glucan exerts multi-layered immunomodulatory effects in tuberculosis through epigenetic reprogramming of innate immune cells, receptor-mediated activation pathways, cytokine modulation, and improved granuloma dynamics. These mechanisms position β-glucan as a promising host-directed therapeutic candidate aimed at strengthening anti-mycobacterial immunity while potentially reducing disease severity and recurrence.

### 4.4 Potential adverse immune activation and safety considerations

While β-glucan–mediated immune activation offers promising host-directed therapeutic potential, excessive stimulation of innate immune pathways may carry potential risks. β-glucan enhances production of pro-inflammatory cytokines such as IL-1β and TNF-α through activation of Dectin-1 and NF-κB signaling pathways (41–46, 64). Although these cytokines are essential for mycobacterial control, their dysregulated or sustained overproduction has been associated with immunopathology and tissue damage in tuberculosis (65, 66).

In pulmonary tuberculosis, excessive inflammatory responses contribute to lung tissue destruction, caseation necrosis, and impaired granuloma integrity (67). Hyperactivation of macrophages and exaggerated Th1 responses may exacerbate local inflammation, potentially worsening disease pathology rather than improving bacterial clearance (67, 68). Therefore, while trained immunity enhances antimicrobial defense, uncontrolled amplification of inflammatory cascades could theoretically destabilize granulomatous architecture and increase pulmonary injury. Additionally, long-term epigenetic reprogramming induced by β-glucan may sustain heightened inflammatory responsiveness beyond the acute phase of infection (46, 69). In individuals predisposed to chronic inflammatory or autoimmune conditions, such persistent activation could pose safety concerns. These considerations highlight the importance of dose optimization, controlled delivery strategies, and careful patient selection in future clinical applications (7, 70). Thus, while β-glucan represents a promising immunomodulatory adjunct in tuberculosis management, its therapeutic application must ensure a balanced immune response that maximizes antimicrobial efficacy without triggering pathological hyperinflammation.

## 5 Immunological responses and role of T-cells in tuberculosis

Following *M. tuberculosis’s* spread to the lymphatic system, the T cell-mediated immune action starts. Antigen-specific T cells get activated and expand before migrating to the diseased lungs wherein they form granulomatous vesicles alongside other leukocytes. There are numerous kinds of T helper cells (including Th1, Th2, Th17, and regulatory T cells) at the sites of disease; nevertheless, the Th1 fraction is traditionally linked to impeded Mycobacterium TB development and dispersal (71).

### 5.1 Th1

**Figure 3:**
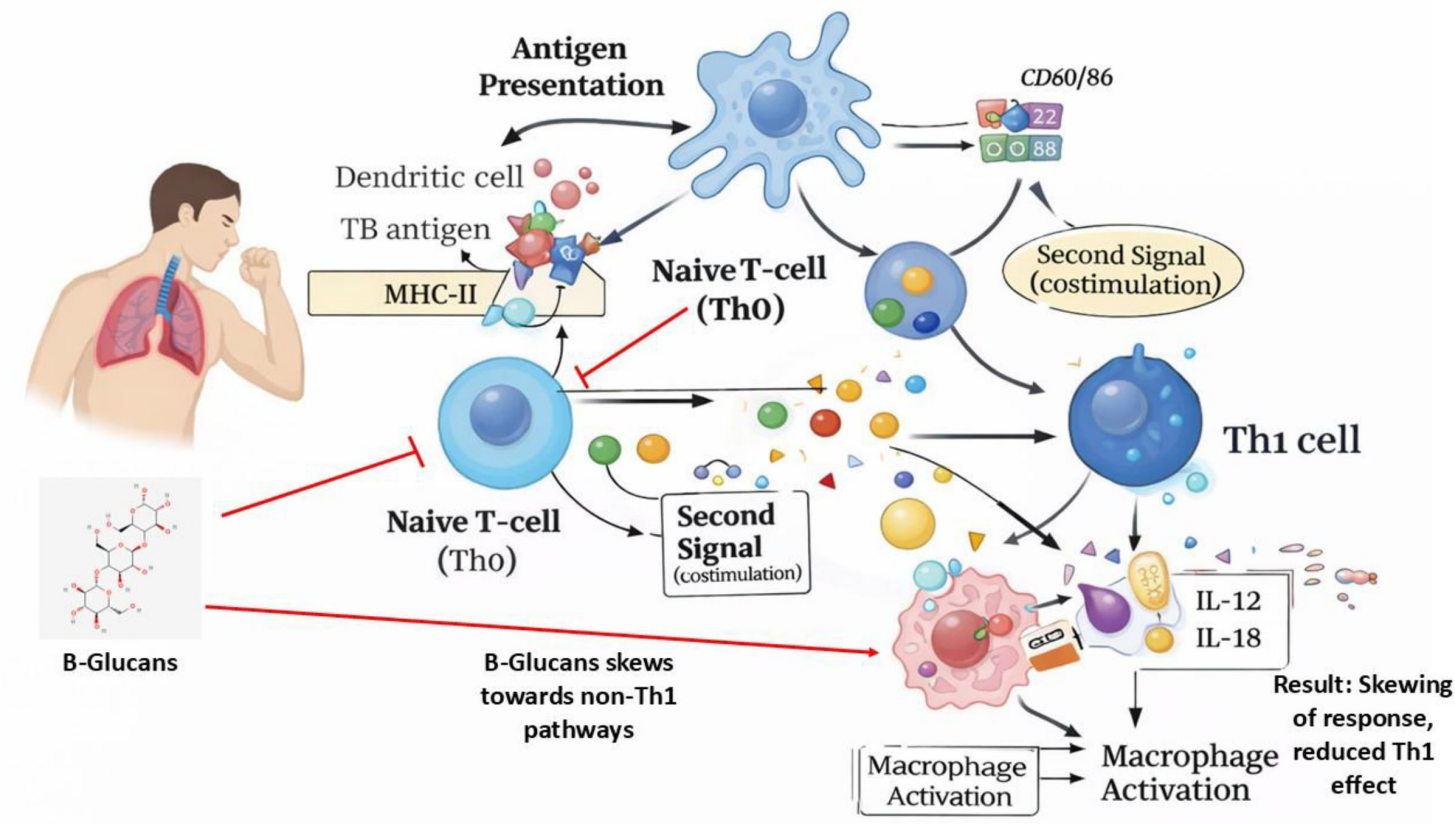
Mechanistic overview of T-helper cell responses and the inhibitory impact of β-glucans during M. tuberculosis infection. While dendritic cells typically present TB antigens to Naive T-cells (Th0) via MHC-II and costimulatory signals (CD80/86) to drive protective Th1 differentiation and macrophage activation, the presence of β-glucans disrupts this pathway. β-glucans inhibit antigen presentation and co-stimulation, skewing the immune response toward non-Th1 pathways. This interference results in reduced Th1 effector functions and impaired macrophage activation, hindering pathogen clearance.

Due to its activation of signaling networks, such as the iNOS cascade and induction of the phase of phagosome deflation and remodeling as well as autophagy, IFN-γ, the primary cytokine of the Th1 profile, promotes macrophage microbicidal capabilities. CD4+ T cells are the major generator of IFN-γ, which is what keeps *M. tuberculosis* in check. Although it is believed that CD8+ T cells, NK cells, T cells, and CD-1-constrained T cells all play important roles in the production of that cytokine, neither of them can compensate for the absence of CD4+ T lymphocyte cells (72). Considering IFN-γs crucial involvement in the battle against *M. tuberculosis*, several experiments have suggested that IFN-gamma’s generation is insufficient to halt the disease’s development. The majority of individuals with chronic Tuberculosis are capable of activating M. tuberculosis-specific IFN-producing T cells at the region of inflammation. It has been demonstrated that patients with stronger reactions have a greater likelihood of developing an active illness than those with lesser reactions (31). Additionally, it has been observed that in people with long-term illnesses, pulmonary parenchyma tissue is drawn to and maintained by T-lymphocytes that are specific for the Mtb antigen. Contrarily, the severity, and persistence of chronic Tuberculosis are correlated with dramatically decreased IFN-γ and IFN-γR signaling. Therefore, suppressed host tolerance in severe TB may be caused by lower frequencies of TNF-α and IFN-γ production as well as IFN-R signaling. (**Figure 3**) (73).

### 5.2 Th2

In test organisms or individuals with tuberculosis, the function of Th2 cytokines, the traditional adversaries of the Th1 profiles, is not entirely understood. Although such cytokines may have an impact on how *M. tuberculosis* avoids the immune system, it is unclear whether they directly contribute to tuberculosis recurrence or not. The explanation of how important autophagy is for defense against M. tuberculosis (44, 74) supported the idea that Th2 cytokines have a role in TB vulnerability. The autophagic mechanism is recognized to be inhibited by Interleukin-4 and Interleukin-13 cytokines (75), which affects antigen distribution, T cell homologous growth, and ultimately the formation of granulation tissue in TB (76). Nevertheless, it is still unknown, that whether the development of Th2 cytokines is the key factor leading to the revival of TB or only an effect of the current infection’s development (67).

### 5.3 T regulatory

When contrasted with the persistence of the pathogen itself, immune-mediated tissue destruction can cause more harm to the host in recurrent sinusitis, such as tuberculosis (TB). As a result, it’s critical to stimulate systems that counteract proinflammatory immune reactions and stop the negative consequences of high inflammation. By generating immunosuppressive cytokines including IL-10, IL-35, and TGF-β, and via actively associating with surrounding cells by blocking helix components, regulatory T cells control inflammation and restrain immune activities (76). Treg cells continue to be a divisive topic when it comes to TB, particularly human illness. According to several research, patients with higher serious chronic illnesses have a greater role of Treg cells, and the loss of these cells leads to the generation of elevated amounts of IFN-γ (77). By reducing IFN-gamma production despite continuing to cause the build-up of polymorphonuclear leukocytes at pulmonary locations, regulatory T cells may also play a significant part in the recurrence of dormant TB and the emergence of chronic tuberculosis (75). Furthermore, according to one research, chronic lung TB is linked with a concurrent rise in the frequency of CD4+ CD25+ and CD4+ CD25+ Foxp3+ cells, but the reduction of CD4+ CD25+ cells has no impact on the microbial infestation or infection-related pulmonary impairment (78, 79).

### 5.4 T17

Whenever naive T cells are triggered in the existence of TGF- β, and Interleukin-6, STAT-3 is activated, which controls the development of such cells into Th17 cells. This in return stimulates the release of the pro-inflammatory cytokine mediators IL-17 and IL-22 as well as upregulation of the transcription factor RORγt (67, 80, 81). The immunoregulatory involvement of the Th17 strain is further strengthened by the activation of IL-21 regulated by IL6. TGF-β is recognized to be overproduced throughout TB and to be present at regions of active M. tuberculosis infection (82), implying that in parallel to its well-known immunomodulatory function, it may also be involved in the development of Th17 cells.

### 5.5 Effect of IL-10 and tuberculosis

Research has shown that the resurgence of TB is more frequently caused by an old illness than by a recent one, supporting the idea that the anti-tuberculosis immunity developed after therapy leads to therapeutic improvement but not to a complete cure. This feature appears to be essential because immune responses controlled by both regulatory and effector processes are necessary to enable the diseased individual to beat off the bacilli without experiencing severe lung impairment or passing away. There are various ways to prevent immune-biological agents, including ones made possible by Foxp3+ regulatory T cells and IL-10 (75). Multiple procedures are in place to eliminate these conditions since the effect of unrestrained TNF-α and IFN-γ can be harmful to the person in situations of invasion or pathogenic implantation, including M. tuberculosis infection. Contradictory to basic assumptions, it has been demonstrated that Th1 and Th9, Th17, and CD8+ T cells, particularly those with long lifespans, can also create IL-10; however, the presence of these multipurpose communities in the perspective of IL-10 in human tuberculosis still requires to be accurately ascertained. Research showed that in individuals who had previously been treated for more than 12 months, showed that the development of a Th1 reaction, which is defined by the elevated release of IFN- γ and TNF-α comes later and is associated with higher IL-10 output (25, 73, 77). Unlike cutaneous leishmaniasis, a protozoan illness, where the Th1 reaction is similarly linked with recovery and the Th1 reaction is triggered soon after therapy, human TB exhibits a gradual emergence of a Th1 sensitivity. Therefore, increased concentrations of IL-10 are crucial for controlling the generation of these pro-inflammatory cytokines, even while the therapeutic curative process continues with the induction of Th1 cytokine generation (IFN-γ and TNF-α). Whether the immune system can eliminate *M. tuberculosis* with the least amount of collateral harm may depend on the equilibrium between such regulators and TNF/IFN (32, 79).

### 5.6 Potential influence of β-glucan on T-cell-mediated immunity in tuberculosis

β-Glucans may also influence adaptive immunity by modulating T-cell responses through activation of innate immune cells (83). Interaction of β-glucans with pattern recognition receptors such as Dectin-1 on macrophages and dendritic cells induces the production of pro-inflammatory cytokines including IL-12, IL-6, and TNF-α. These cytokines promote the differentiation of naïve T cells into Th1 and Th17 subsets, which are critical for protective immunity against *M. tuberculosis* (84). Enhanced Th1 responses increase IFN-γ production, thereby promoting macrophage activation and intracellular killing of *M. tuberculosis*. Additionally, β-glucan-induced activation of antigen-presenting cells may improve CD8+ T-cell cytotoxic responses against infected cells (85).

## 6 Treatment and management of tuberculosis

According to WHO recommendations, people with Tuberculosis must receive treatment from the appropriate initiatives using an incorporated, management strategy for disease protection, assessment, treatment, and impact minimization (29, 86, 87). Care for TB should involve collaborative healthcare personnel monitoring and assistance, even during the early stages. Moreover, owing to much more substantial tuberculosis, an impaired immune response, or limited doses of Tuberculosis drugs in the patients, the rates of Tuberculosis treatment failure in patients with this disease (3, 88). As a result, longer treatment times and weight-adjusted or dependent doses of anti-tuberculosis medications may be required. The best strategy for managing Tuberculosis in patients may involve minimizing sulphonyl urea derivatives and managing the condition with nutrition, behavioral changes, and medications. Only through assuring excellent conformance to therapy for tuberculosis, can effective treatment management be attained. Treatment interruptions can promote the formation of drug-resistant, worsen infection and impairment, and make it relatively easy for tuberculosis to spread (89, 90). In addition to conventional antimicrobial therapy, there is increasing interest in host-directed therapies that enhance immune responses against *M. tuberculosis*. One potential approach involves the use of immunomodulatory compounds such as β-glucans as adjunctive agents alongside standard anti-tuberculosis drugs. These compounds may strengthen host immune defenses and improve the ability of immune cells to control mycobacterial infection.

The immune-boosting properties of beta-glucan, which is a component of the cell walls of fungi, yeasts, and cereals, have long been recognized (91). It has recently been demonstrated that beta-glucan is also a potent stimulant of trained immunity. When stimulated in the laboratory with diverse microbiological ligands after being previously liable to beta-glucan, human monocytes show an improved immune function (32, 41, 92). In addition, many researchers found that therapy or treatment with beta-glucan affords macrophage-derived resistance from re-exposure to infections, which could include both bacterial as well as fungal infections (48, 79, 93–95). It seemed previously difficult to determine the principle behind the persistent therapeutic benefits of beta-glucan due to the limited lifetime of myelocytes in blood circulation. However according to a current investigation by Mitroulis et al. (2018) (40), beta-glucan not only triggers trained immunity in developed cell types such as macrophages and monocytes but also modifies the working program of hematopoietic precursors in the bone marrow, which most likely explains the long-term formation of trained myelocytes in the bloodstream. This process, known as emergency or trained myelopoiesis, leads to the generation of functionally reprogrammed innate immune cells with enhanced antimicrobial activity. These cells may exhibit increased cytokine production, improved pathogen recognition, and stronger antimicrobial responses, potentially contributing to improved host resistance against *M. tuberculosis* infection (96, 97). Similar stimulants of trained immunity, including the Bacille Calmette-Guerin (BCG) vaccination and a high-calorie intake, have also been linked to comparable changes at the bone marrow level (98–100).

Through these mechanisms, β-glucans may act as a supportive immunomodulatory intervention that complements conventional antibiotic therapy. By enhancing innate immune responsiveness and promoting trained immunity, β-glucan supplementation could theoretically reduce bacterial burden and improve treatment outcomes when used alongside standard anti-tuberculosis regimens. However, although experimental and immunological studies suggest promising benefits, well-designed clinical trials are still required to determine the efficacy, optimal dosage, and safety of β-glucan as an adjunct therapy for tuberculosis management.

### 6.1 Biomarkers for monitoring treatment response and β-glucan therapy

**Figure 4:**
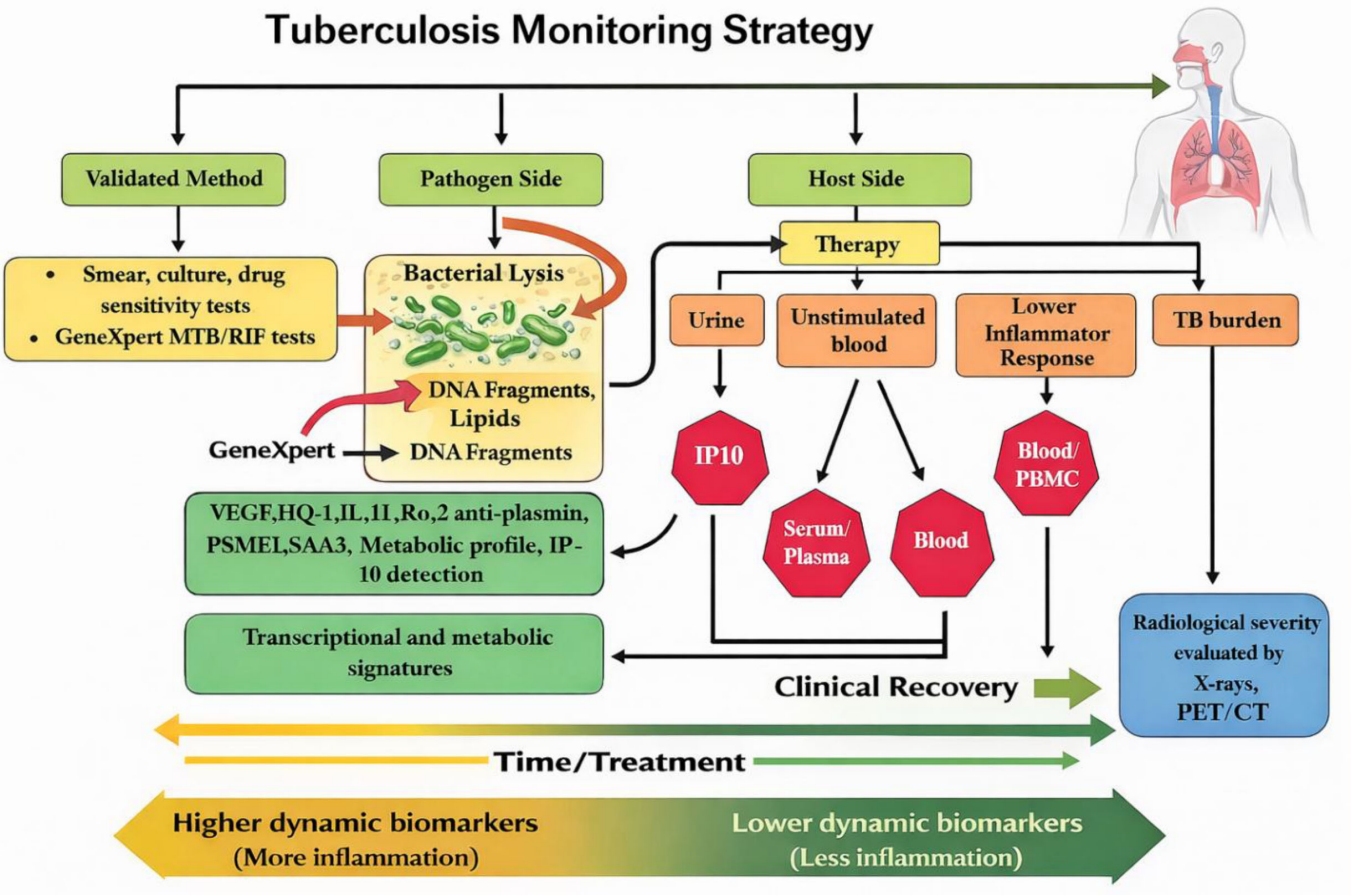
Tuberculosis monitoring strategy illustrating the dynamic relationship between therapy, pathogen clearance, and host biomarker responses during treatment. (TB, Tuberculosis; VEGF, Vascular endothelial growth factor; BAL, Broncholavage; FACS, Fluorescence-activated cell sorting; IL, Interleukin; PSME1, Proteasome activator complex subunit 1; SAA, Serum amyloid A; PET/CP, Positron emission tomography/computed tomography).

Appropriate treatment is the cornerstone of any TB control strategy, and biomarkers that show the onset of a successful course of treatment may make it easier to establish various treatment plans (64, 101). Mtb in sputum, which depends on the presence of necrotic infection foci, is commonly used to diagnose and monitor active TB (102). However, these conventional indicators do not fully reflect the host immune response during treatment. In addition to pathogen-based measures, host immune biomarkers such as cytokine levels (IL-1β, TNF-α, IFN-γ) and T-cell activation markers (CD38, HLA-DR, and Ki-67) have been associated with disease activity and treatment response (54, 103–105). These markers may provide insights into immune restoration and bacterial clearance during therapy (**Figure 4**). Since β-glucans modulate innate immune signaling pathways and cytokine production, monitoring host inflammatory markers and immune activation profiles could help evaluate the immunological impact of β-glucan supplementation during tuberculosis treatment (41, 52, 53). Therefore, while conventional biomarkers remain important for assessing treatment outcomes, immune biomarkers may be useful in evaluating host-directed therapeutic strategies aimed at enhancing anti-mycobacterial immunity. Several steps can be implemented within the end-TB strategic plan to minimize the load of TB, including monitoring, initial identification, sufficient assistance systems, providing care to patients with this disease, investigations, and development (106, 107). Prevention involves tackling the innate causes of TB. Additionally, the frequent interactions with medical care professionals during TB treatment present a tremendous possibility for health counseling and education for improved diabetes prevention (21, 108–110).

### 6.2 Research gaps

Although increasing attention has been directed toward host genetic susceptibility and immune-modulatory compounds in TB, significant knowledge gaps remain. It will be important to further investigate genetic traits that have not yet been fully characterized in order to obtain a clearer understanding of TB pathogenesis. To date, no conclusive association has been established between specific alleles or gene expression patterns and the precise etiology or progression of TB in diverse human populations. This indicates a need for broader genomic and transcriptomic investigations to identify novel host factors that influence susceptibility, disease severity, and treatment response. Another emerging area of interest is the immunomodulatory role of β-glucan. Some studies suggest that β-glucan can induce functional reprogramming of human monocytes, leading to a distinct interleukin-1-mediated inflammatory profile and enhanced anti-mycobacterial activity. These responses are believed to strengthen host immune defense while simultaneously regulating excessive inflammation, potentially reducing the risk of TB development. However, the underlying molecular mechanisms governing β-glucan–mediated immune modulation in TB remain poorly understood. Further, current studies on β-glucan are largely limited to *in vitro* macrophage models or animal infection systems, which may not fully represent the complexity of human TB infection. Structural heterogeneity among β-glucans derived from different sources (such as yeast, fungi, and cereals) also leads to variability in receptor interactions and immune responses. The lack of standardized extraction methods, dosing strategies, and well-designed clinical trials further limits the translation of these findings into therapeutic applications. Therefore, future research integrating advanced molecular techniques, immunogenomics, and clinical studies is essential to elucidate the mechanisms of β-glucan action and to better understand the genetic and immunological dynamics underlying TB spread, diagnosis, and disease progression.

## 7 Conclusion

Beta-glucan aids in the altering of monocytes and macrophages, which helps suppress the Mtb infection and contributes to improving the host defense against the pathogen. Beta-glucan has been found to promote trained immunity, leading to long-lasting functional modifications in monocytes and macrophages, resulting in enhanced resistance to microbial infections. In addition, beta-glucan modifies histones at gene promoters in human monocytes, leading to increased release of proinflammatory cytokines and suppression of Mtb development. Through these mechanisms, β-glucan strengthens innate immune responses and supports the host’s ability to control tuberculosis infection. Administering β-glucan might enhance anti-tuberculosis vaccines and treat mycobacterial infections in a unique way. These immunomodulatory properties suggest that β-glucan could potentially be used as a host-directed therapeutic or adjunct intervention alongside conventional anti-tuberculosis treatments to improve immune responsiveness against Mtb. Furthermore, additional investigation into the immunological activity and mechanism of action of beta- glucans is likely to expand their potential applications. As a result, Beta-glucan reveals promise as an essential therapeutic strategy for tuberculosis by improving immunomodulatory responses and supporting host defense. However, further research is needed to fully understand the underlying mechanism and therapeutic potential of beta-glucan in tuberculosis.
